# Effect of Forest Walking on Autonomic Nervous System Activity in Middle-Aged Hypertensive Individuals: A Pilot Study

**DOI:** 10.3390/ijerph120302687

**Published:** 2015-03-02

**Authors:** Chorong Song, Harumi Ikei, Maiko Kobayashi, Takashi Miura, Masao Taue, Takahide Kagawa, Qing Li, Shigeyoshi Kumeda, Michiko Imai, Yoshifumi Miyazaki

**Affiliations:** 1 Center for Environment, Health and Field Sciences, Chiba University, Kashiwa, Chiba 277-0882, Japan; E-Mails: crsong1028@gmail.com (C.S.); ikei.harumi@gmail.com (H.I.); 2 Department of Hygiene and Public Health, Nippon Medical School, Bunkyo-Ku, Tokyo 113-8602, Japan; E-Mails: mk831111@nms.ac.jp (M.K.); qing-li@nms.ac.jp (Q.L.); 3 Agematsu Town Office Industry & Tourism Department, Kiso, Nagano 399-5601, Japan; E-Mail: syoukan@town.agematsu.nagano.jp; 4 Agematsu Town Office, Kiso, Nagano 399-5603, Japan; E-Mail: taue@town.agematsu.nagano.jp; 5 Forestry and Forest Products Research Institute, Tsukuba City, Ibaraki Prefecture 305-8687, Japan; E-Mail: kagawa@ffpri.affrc.go.jp; 6 Nagano Prefectural Kiso Hospital, Nagano 397-8555, Japan; E-Mail: kumeda-shigeyoshi@pref-nagano-hosp.jp; 7 Le Verseau Inc., Setagaya-ku, Tokyo 156-0051, Japan; E-Mail: leverseau@mvb.biglobe.ne.jp

**Keywords:** forest therapy, urban environment, walking, hypertension, middle-aged individuals, preventive medicine, heart rate variability, heart rate, semantic differential method, profile of mood state

## Abstract

There has been increasing attention on the therapeutic effects of the forest environment. However, evidence-based research that clarifies the physiological effects of the forest environment on hypertensive individuals is lacking. This study provides scientific evidence suggesting that a brief forest walk affects autonomic nervous system activity in middle-aged hypertensive individuals. Twenty participants (58.0 ± 10.6 years) were instructed to walk predetermined courses in forest and urban environments (as control). Course length (17-min walk), walking speed, and energy expenditure were equal between the forest and urban environments to clarify the effects of each environment. Heart rate variability (HRV) and heart rate were used to quantify physiological responses. The modified semantic differential method and Profile of Mood States were used to determine psychological responses. The natural logarithm of the high-frequency component of HRV was significantly higher and heart rate was significantly lower when participants walked in the forest than when they walked in the urban environment. The questionnaire results indicated that, compared with the urban environment, walking in the forest increased “comfortable”, “relaxed”, “natural” and “vigorous” feelings and decreased “tension-anxiety,” “depression,” “anxiety-hostility,” “fatigue” and “confusion”. A brief walk in the forest elicited physiological and psychological relaxation effects on middle-aged hypertensive individuals.

## 1. Introduction

During the seven-million-year history of humans [1], they have lived in natural environments; thus, they experienced a drastic change when they began living in urban environments. Rapid urbanization and artificialization have affected the environment by increasing traffic along with air and water pollution, while decreasing the amount of available agricultural land and open spaces [2]. These environmental changes, especially climate changes, threaten human health and quality of life (QOL) [2,3,4,5]. Furthermore, the rapid development of information technology has increased what Brod describes as “technostress” [6], a modern disease of adaptation caused by unhealthy coping mechanisms for dealing with new computer technologies. When combined, these factors can severely affect humans. Several studies have reported that urban environments are stressful [7,8,9] and are associated with increasing mortality rates [10].

In our stressful modern age, the relaxing effects of a natural environment are very important. As our interest in improving health and QOL has increased, more attention has been focused on the role of nature in promoting human health and well-being. In particular, a great deal of attention is focused on the therapeutic effects of the forest environment or “forest therapy.” Forest therapy uses the medically proven effects of walking in a forest and observing the environment to promote feelings of relaxation and improve both physical and mental health.

Many studies have demonstrated that a forest environment can have positive physiological and psychological effects [11,12,13,14,15,16,17,18,19,20,21,22,23,24,25,26,27]. When compared with an urban environment, viewing forest scenery or walking in forests can decrease cerebral blood flow in the prefrontal cortex [11], reduce blood pressure [12,13,14,15] and pulse rate [12,13,14,16,17], increase parasympathetic nerve activity [12,14,15,16,17,18,19], suppress sympathetic nerve activity [12,14,15,17,18,19], and decrease salivary cortisol concentrations of stress hormones [11,12,13,15,16,17]. In addition, a previous study reported that visiting a forest enhanced natural killer cell activity and improved immune function [20], and these effects continued for up to 1 month [21,22]. With regard to the psychological effects, several questionnaire-based studies reported that people who are in a forest environment experience positive feelings, which they describe as “comfortable”, “soothed” and “natural” [11,12,13,14,17], as well as an improved mood and cognitive functioning [15,17,18,19,23,24,25,26,27].

Forest therapy has recently attracted attention as a preventive or alternative therapy [28,29], and its effects have been studied in elderly individuals and patients with reversible diseases. Lee and Lee [30] demonstrated that walking in a forest for 1 h improves arterial stiffness and pulmonary function in elderly women. Otsuka *et al.* [31] clarified that forest walking decreased blood glucose levels in patients with non-insulin-dependent diabetes mellitus. Other findings have indicated that cognitive behavioral therapy conducted in a forest environment was more successful in achieving depression remission than psychotherapy conducted in a hospital [32].

Several studies have demonstrated positive effects in hypertensive individuals. Mao *et al.* [33] reported that a seven-day forest-bathing trip reduces blood pressure and decreases pathological indicators of cardiovascular disease. Sung *et al.* [34] demonstrated that a frequent and eight weeks’ forest therapy program based on cognitive behavioral therapy can reduce salivary cortisol levels and improve QOL in hypertensive patients. However, to the best of our knowledge, there are no evidence-based research studies that have used indices of autonomic nerve system activity to clarify the acute response of exposure to a forest environment.

Therefore, the purpose of the present study was to clarify the acute response of forest walking on autonomic nerve activity. We used heart rate variability (HRV) [35,36] and heart rate to measure autonomic responses and then compared these responses among middle-aged hypertensive individuals who walked in a forest and an urban environment.

## 2. Materials and Methods

### 2.1. Participants

Twenty Japanese men (mean age, 58.0 ± 10.6 years; mean body mass index, 23.4 ± 3.3 kg/m^2^) participated in the experiment. The participants’ information and characteristics are shown in Table 1. Participants who were taking medication for chronic conditions such as diabetes, hyperlipidemia, and hypertension were excluded. Among these 20 participants, five had a high-normal blood pressure (systolic 130–139 mmHg or diastolic 85–89 mmHg) that was considered in the higher range of prehypertension. Of the remaining 15 participants, 10 had hypertension stage 1 (systolic 140–159 mmHg or diastolic 90–99 mmHg) and five had hypertension stage 2 (systolic 160–179 mmHg or diastolic 100–109 mmHg). Furthermore, for the classification, the values measured in the morning (8:30–8:45) of the first experimental day were used.

Before the experiment, the participants were fully informed about the study aims and procedures; and after receiving a description of the experiment, they signed an agreement to participate in the study. Consumption of alcohol and tobacco was prohibited and consumption of caffeine was controlled during the study period. All subjects gave their informed consent for inclusion before they participated in the study. The study was conducted in accordance with the Declaration of Helsinki, and the protocol was approved by the Ethics Committees of the Nagano Prefectural Kiso Hospital, Japan and of the Center for Environment, Health, and Field Sciences, Chiba University, Japan (Project identification code number: 5).

**Table 1:** Participant demographics.

Parameter	Value (Mean ± Standard deviation)
Total sample number	20
Sex	Male
Age (years)	58.0 ± 10.6
Height (cm)	167.9 ± 6.2
Weight (kg)	66.1 ± 10.6
BMI (kg/m^2^)	23.4 ± 3.3
SBP (mmHg)	151.2 ± 17.9
DBP (mmHg)	90.7 ± 5.0

### 2.2. Experimental Sites

The field experiments were conducted in a coniferous forest that included many Japanese cypress trees (Akasawa Shizen Kyuyourin; Akasawa natural recreation forest) and was located in Agematsu town of Nagano Prefecture situated in central Japan (hereafter referred to as the forest area). An urban area in Ina City of Nagano Prefecture was selected as the control site (hereafter referred to as the urban area). The weather was sunny on the days of experiments. In the forest area, the average temperature was 21.4 ± 1.2 °C with an average humidity of 82.3 ± 4.8%, whereas in the urban area, the average temperature was 28.1 ± 1.1 °C with an average humidity of 61.9 ± 4.5%.

### 2.3. Physiological Indices

HRV and heart rate, which were used to quantify autonomic nervous system responses, were measured using a wearable electrocardiogram sensing system (myBeat; Union Tool, Co., Tokyo, Japan). Frequency spectra were generated using an HRV software tool (MemCalc/Win; GMS, Tokyo, Japan) [37]. For real-time HRV analysis by the maximum entropy method, interbeat (R-R) intervals were obtained continuously. In this study, two broad HRV spectral components were calculated: low frequency (LF; 0.04–0.15 Hz) and high frequency (HF; 0.15–0.40 Hz). The HF component is an estimate of parasympathetic nerve activity, whereas the LF/HF ratio is an estimate of sympathetic nerve activity [35,36]. To normalize HRV parameters across participants for the analysis, we transformed the values using the natural logarithm [38].

### 2.4. Psychological Indices

The participants answered two questionnaires to investigate psychological responses. The modified semantic differential (SD) method [39] used three pairs of adjectives on thirteen scales, including “comfortable to uncomfortable”, “relaxed to awakening” and “natural to artificial”. The Profile of Mood State (POMS) [40,41,42] scores were determined for the following six subscales: “tension-anxiety”, “depression”, “anger-hostility”, “fatigue”, “confusion” and “vigor”. We used a short version of the POMS that included 30 questions in order to decrease the participants’ burden.

### 2.5. Experimental Design

We performed a within-subject experiment. The 20 participants were randomly assigned to two groups of 10 each that participated in the experiment over two consecutive days. On the first day, one group traveled to the forest area and the other traveled to the urban area by car (about 45 min). On the second day, the groups switched walking courses to eliminate an order effect.

**Figure 1:**
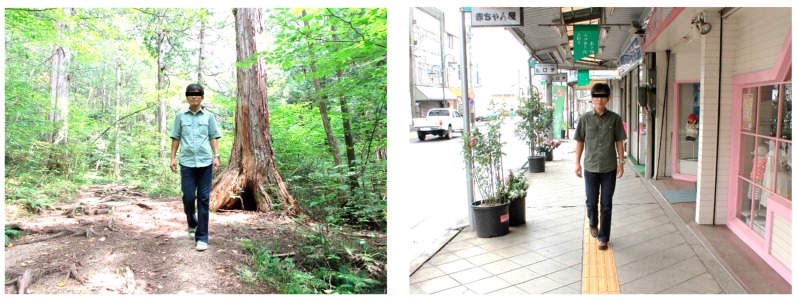
Experimental sites.

The participants moved within their respective experimental site once they arrived. After resting for 10 min, the participants were instructed to walk a predetermined course. An experimenter guided the participants along the course; the duration of each walk was 17 min (Figure 1), and the two experimenters leading the courses walked at almost the same speed. The course duration and walking speed of both the experimenters were set to be the same for both the forest and urban areas. The walking course in the forest area was mostly flat, except for a small slope (3.25%) in the first 6 min of the course, whereas that of the urban area was flat. The participants walked the two courses at approximately the same time of day (10:30–11:10) to eliminate the influence of diurnal changes on physiological rhythms.

HRV and heart rate data were collected at 1-min intervals and then averaged over the entire 17-min course. We then compared these average values between sites. Energy expenditure for walking was also assessed (Lifecorder GS4; Suzuken Co., LTD., Chiba, Japan). The participants answered the two questionnaires after completing each course.

### 2.6. Statistical Analyses

Physiological data of 19 participants were used for analysis because of errors in data collection for one participant. We used the paired t-test to compare the mean HRV and heart rate between the two walking sites. We used the Wilcoxon signed-rank test to analyze differences the psychological indices completed after walking in each environment. All statistical analyses were performed using SPSS 20.0 (IBM Corp., Armonk, NY, USA). In all comparisons, a *p*-value of <0.05 was considered statistically significant. One-sided tests were used for both comparisons because our hypothesis was that elderly hypertensive individuals would also be relaxed after walking in a forest.

## 3. Results

We confirmed there were no significant differences in the energy expenditure between the two environments (forest, 1.99 kcal/min; urban, 2.03 kcal/min, *p* > 0.05). However, the participants showed significant differences in their physiological and psychological responses for the 17-min walk in the forest and the urban areas.

**Figure 2:**
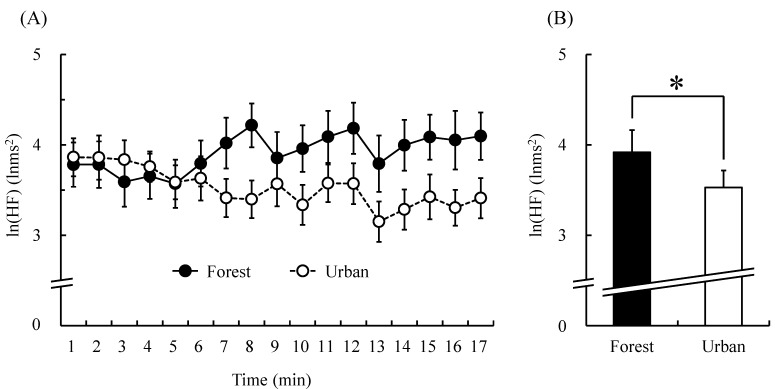
ln(HF) value of heart rate variability during the forest and urban walk. (**A**) Changes in each 1-min average ln(HF) value over the 17-min walk. (**B**) Overall mean ln(HF) values. *N* = 19, mean ± standard error. * *p* < 0.05, paired *t*-test.

Figure 2 shows the natural logarithm of HF component ln(HF), which is an estimate of parasympathetic nerve activity. In the 1-min segment analysis, most ln(HF) values were higher when participants walked in the forest than when they walked in the urban area, except during the first 4-min period (Figure 2A). The mean ln (HF) over the entire walking period was significantly higher in forest walking than in urban walking (forest, 3.9 ± 0.2 lnms^2^; urban, 3.5 ± 0.2 lnms^2^; *p* < 0.05, Figure 2B). In contrast, there was no significant difference between the two environments for the natural logarithm of LF/HF (ln(LF/HF)), an estimate of sympathetic nerve activity.

**Figure 3:**
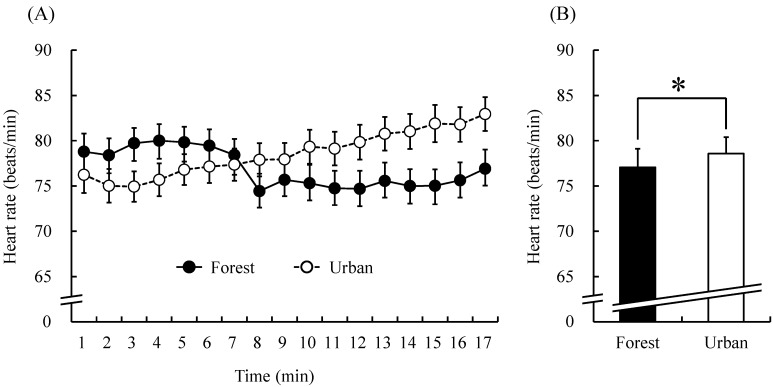
Heart rate during the forest and urban walk. (**A**) Changes in each 1-min heart rate value over the 17-min walk. (**B**) Overall mean heart rates. *N* = 19, mean ± standard error. * *p* < 0.05, paired *t*-test.

Heart rate values were lower in forest walking than in urban walking, except during the first 6-min period (Figure 3A). The mean heart rate during the entire 17-min walk was significantly lower when participants walked in the forest area than when they walked in the urban area (forest, 77.1 ± 2.0 bpm; urban, 78.6 ± 1.8 bpm; *p* < 0.05, Figure 3B).

**Figure 4:**
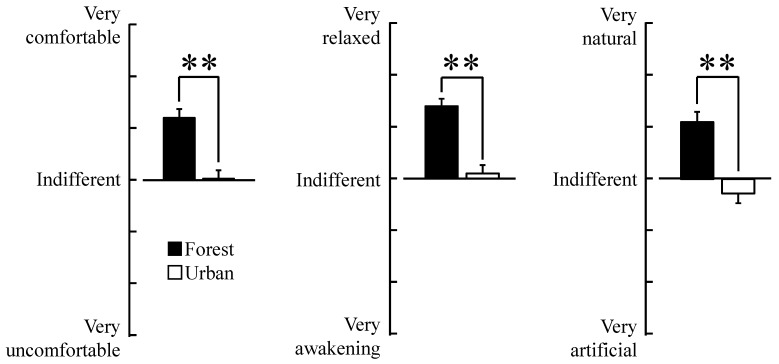
Comparison of “comfortable,” “relaxed,” and “natural” feeling scores between the two environments. *N* = 20, mean ± standard error. ** *p* < 0.01, Wilcoxon signed-rank test.

**Figure 5:**
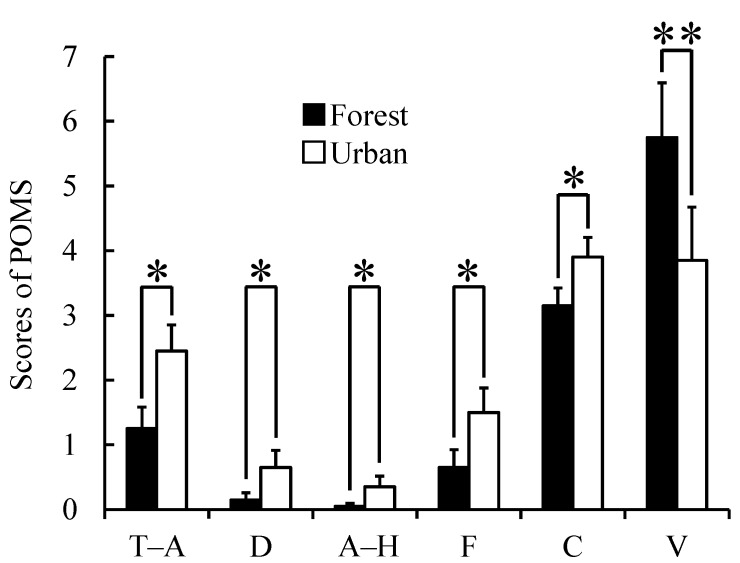
Comparison of Profile of Mood State (POMS) scores between the two environments. T–A: tension–anxiety; D: depression; A–H: anger–hostility; F: fatigue; C: confusion; V: vigor. *N* = 20, mean ± standard error. * *p* < 0.05, ** *p* < 0.01, Wilcoxon signed-rank test.

Our analysis of the participants’ responses to the two questionnaires, the SD method and the POMS scores, revealed differences in psychological responses between the two environments. Participants felt more “comfortable”, “relaxed” and “natural” when they walked in the forest area than in the urban area (*p* < 0.01, Figure 4). We also observed differences in the POMS test in which scores for the negative subscales of “tension–anxiety”, “depression”, “anger-hostility”, “fatigue” and “confusion” were significantly lower after walking in the forest area than after walking in the urban area (*p* < 0.05, Figure 5). Conversely, the positive mood state “vigor” was significantly higher after walking in the forest area than after walking in the urban area (*p* < 0.01, Figure 5).

## 4. Discussion

A short walk in a forest can have significant physiological and psychological effects on middle-aged hypertensive individuals. Compared with walking in the urban environment, walking in the forest environment significantly increased parasympathetic nerve activity and significantly decreased heart rate. These results are consistent with those from previous studies that examined physiological responses to a forest environment in young adults [12,14,15,16,17,18,19]. HRV responses are often detected during relaxed states such as during rest [35], a massage [43,44], or after performing yoga [45]. Therefore, we concluded that participants who walked in the forest were in a physiologically relaxed state.

On the other hand, we observed the reverse in our analysis of ln(HF) and heart rate for the 1-min segments. We do not know the exact reason for this difference; however, because heart rate increases during walking and running, especially uphill [46,47,48], we suppose these physiological responses resulted from the small slope at the beginning of the forest area course. The slope was characterized by a forest environment. We believe that if this feature is used well, it will be of great merit to forest therapy programs.

In the questionnaires, the participants reported that they felt more “comfortable”, “relaxed” and “natural” after walking in the forest. In addition, negative emotions such as “tension-anxiety,” “depression”, “anger-hostility”, “fatigue” and “confusion” as well as the positive emotion of “vigor” improved significantly after walking in the forest. Our findings of the psychological benefits of walking in a forest are partly consistent with previous findings [11,12,13,14,15,18,23]. In the modern age, the importance of mental health has increased [49]. The psychological benefits of a forest environment may play a very important role in improving mental stress.

Furthermore, physical condition such as the average temperature (forest: 21.4 ± 1.2 °C, urban: 28.1 ± 1.1 °C) and humidity (forest: 82.3 ± 4.8%, urban: 61.9 ± 4.5%) was significantly different (*p* < 0.01 by unpaired t-test). Park *et al.* [23] examined the relationship between psychological responses to forest and urban areas and the physical variables of these environments. As a result, the psychological responses to physical environments were also significantly related to air temperature, relative humidity, radiant heat, wind velocity, PMV, and PPD. It is considered that different physical condition is one of the reasons for differences in physiological and psychological responses in the present results.

Walking is a simple, accessible, and cost-effective method to improve physical health, and this has been clarified in previous studies [50,51]. Iwane *et al*. [50] reported that walking at least 10,000 steps per day can lower blood pressure and suppress sympathetic nerve activity in hypertensive patients. Williams and Thompson [51] demonstrated that equivalent energy expenditures in walking and running could produce similar risk reductions for hypertension, hypercholesterolemia, and diabetes mellitus. However, it is not yet elucidated whether such effects can be attributed to differences in the walking environment.

The present findings suggest that these effects can differ with the environment. The present findings also clearly demonstrate that in middle-aged men, a brief walk in the forest was associated with relaxing physiological and psychological effects. However, this study had a few limitations. To generalize the findings, it is necessary to consider the following: First, these results cannot be extrapolated to the female population and people of different age groups. Further studies on a large sample including various subject groups are required. Second, the present study only used HRV and heart rate. For the overall discussion, future studies should be assessed to determine the effects of forest environment using other physiological indices, such as brain activity, autonomic nervous activity and endocrine activity.

## 5. Conclusions

Regarding the physiological and psychological effects of a brief walk in the forest environment for middle-aged individuals with hypertension, our study findings revealed the following: (1) a significant increase in parasympathetic nerve activity; (2) a significant decrease in heart rate; (3) a significant increase in “comfortable,” “relaxed,” and “natural” feelings assessed by the modified SD method combined with significant improvements in “tension-anxiety”, “depression”, “anger-hostility”, “fatigue”, “confusion” and “vigor” assessed by the POMS. In conclusion, walking in a forest induced physiological and psychological relaxation.
